# A case of thoracic aortic aneurysm discovered following poor ventilation during induction of anesthesia

**DOI:** 10.1186/s40981-025-00827-3

**Published:** 2025-10-31

**Authors:** Mahiro Isogai, Ko Ishikawa, Ryosuke Funabiki, Takashi Suto, Shigeru Saito

**Affiliations:** 1 Department of Anesthesiology, Isesaki Municipal Hospital, 12-1 Tsunatorihonmachi, Isesaki, Gunma 372-0817 Japan; 2 Department of Anesthesiology, Gunma University Hospital, 3-39-15 Showa, Maebashi, Gunma 371-8511 Japan; 3 Department of Anesthesiology, Saiseikai Utsunomiya Hospital, 911-1 Takebayashi, Utsunomiya, Tochigi 321-0974 Japan; 4 Gunma University Hospital, 3-39-15 Showa, Maebashi, Gunma 371-8511 Japan

**Keywords:** Tracheal stenosis, Thoracic aortic aneurysm, Ventilation difficulties, Anesthesia induction

## Abstract

Several cases of tracheal stenosis due to thoracic aortic aneurysm have been reported. However, cases of aortic aneurysm discovered following ventilation difficulties immediately after the induction of general anesthesia are rare. The patient, a 90-year-old woman with a history of ascending aortic replacement for a thoracic aortic aneurysm, was scheduled for sternum infection debridement. During induction, adequate ventilation could not be achieved despite the use of positive pressure, even after tracheal intubation. Bronchoscopy showed tracheal compression, which was not apparent in preoperative evaluations. CT imaging on the day demonstrated that the compression was caused by a thoracic aortic aneurysm. A thoracic endovascular aneurysm repair was performed successfully, and the tracheal compression improved afterwards. Compression from outside the trachea should be considered as a potential cause of sudden ventilation difficulties. Particularly in patients with a history of aortic surgery, additional preoperative assessments should be considered.

## Background

Although there are several reported cases of tracheal stenosis caused by a thoracic aortic aneurysm and discovered due to respiratory distress, there are no reported cases of thoracic aortic aneurysm discovered due to ventilation difficulties during induction of anesthesia in patients who have no imaging findings of tracheal stenosis prior to induction of anesthesia. External compression of the trachea by an aortic aneurysm may also be a cause of unexpected ventilation difficulties during induction of anesthesia. Compression from outside the trachea should also be considered as a cause of sudden ventilation difficulties. Particular attention should be taken even in asymptomatic postoperative patients, as in this case.

## Case presentation

A 90-year-old woman (height, 145 cm; weight, 51.4 kg) underwent ascending aortic replacement 3 years ago for a thoracic aortic aneurysm. She had a history of asymptomatic cerebral artery aneurysms, controlled hypertension, and anemia. Three months previously, she had been hospitalized for a positional cough induced in the left lateral position and treated for heart failure.

Due to chronic infection (duration, 1 year) of the subcutaneous tissue above the sternum and closure wires, the patient was scheduled for wound debridement under general anesthesia on day Z. General anesthesia was induced using propofol (1 mg/kg), rocuronium (0.6 mg/kg), and remifentanil (0.2 μg/kg/min). Mask ventilation was started after sleep was confirmed, but a sufficient ventilation volume could not be achieved with a positive pressure of about 20–30 cmH_2_O. Capnometry showed a pressure of 10–15 mmHg. Placement of an oral airway did not improve the ventilation. Endotracheal intubation using a 7.0 mm (ID) tube was then attempted by the anesthesiologist, but the tube could not be placed more than 18 cm from the right oral angle, and it did not produce sufficient ventilation. The anesthesiologist used a video laryngoscope to verify the placement of the tube within the trachea through the vocal cords. The capnography waveforms were very small, suggesting bronchospasm or severe tracheal obstruction. Salbutamol 200 μg, when sprayed intratracheally, did not improve ventilation.

**Fig. 1:**
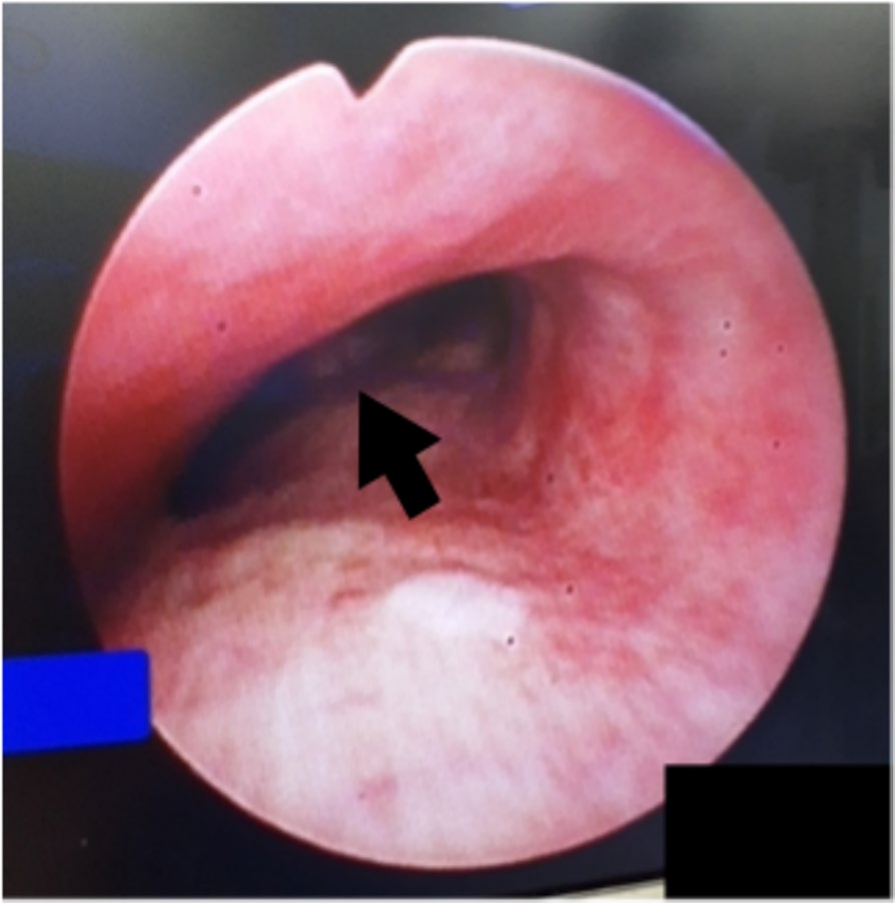
An image obtained during bronchoscopy shows stenosis of the left main bronchus due to an elevated and pulsating membranous portion of the trachea immediately above the bifurcation. Arrow: Carina

Bronchoscopy of the tracheal lumen above the bifurcation revealed elevation and pulsation of the membranous portions of the trachea (Fig. 1). This suggested that ventilation issues might be caused by tracheal stenosis resulting from compression by a thoracic aortic aneurysm. The lowest oxygen saturation (SpO_2_) during the procedure was 81%, but it returned to 100% immediately after sputum was removed from the narrowed trachea. Her blood pressure and heart rate remained within approximately ± 30% of their pre-anesthesia values.

Given the risk of the patient's overall condition deteriorating if the surgery were postponed, surgery was performed as scheduled under general anesthesia. She was then transported to the intensive care unit (ICU) and her blood pressure was strictly controlled by intravenous nicardipine.

**Fig. 2:**
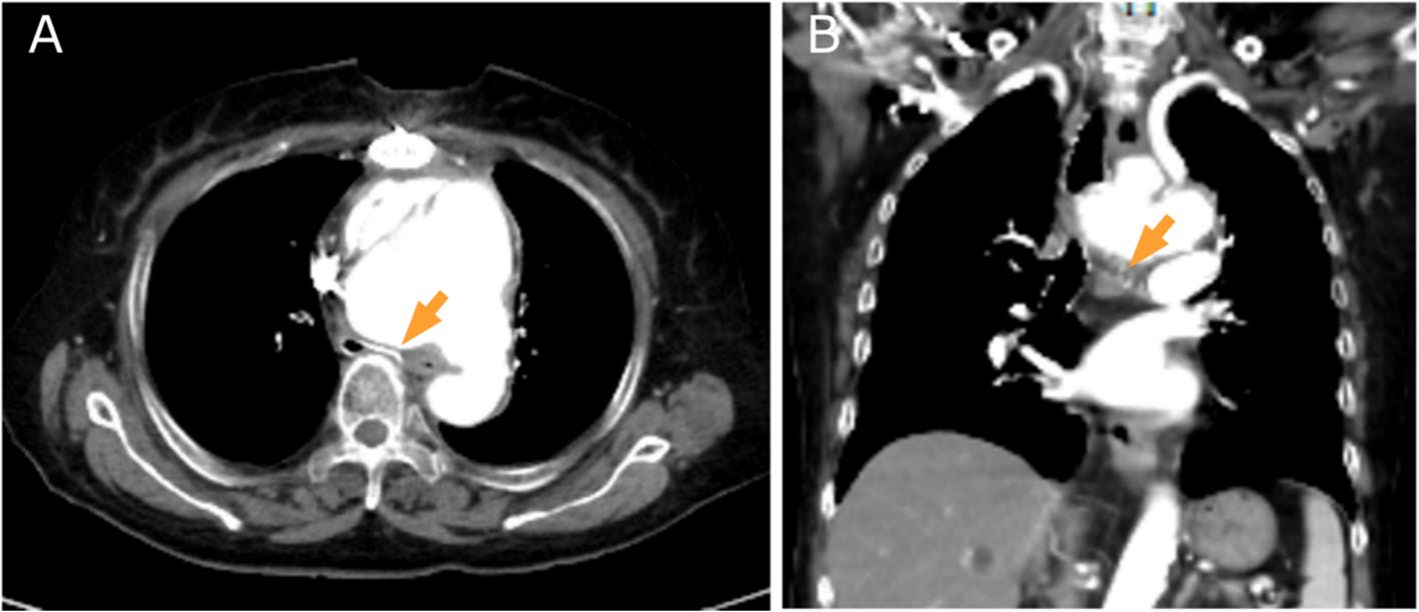
CT image of the chest (**A** transverse plane, **B** coronal plane) on day Z + 1. The images show dilation of the aortic arch (maximum diameter: 86 mm). The trachea is compressed and narrowed (arrows) by the aneurysm and the vertebral body above the bifurcation

Computed tomography (CT) of the chest on day Z + 1 revealed dilation of the aortic arch (maximum diameter: 86 mm). The trachea was compressed and narrowed by the aneurysm and the vertebral body above the bifurcation. In the supine position, the left main bronchus was severely compressed and narrowed (Fig. 2). As the aneurysm was in a state of imminent rupture, thoracic endovascular aneurysm repair (TEVAR) was scheduled on day Z + 2.

## Second surgery

The patient was in a supine position on a bed slightly tilted to the right. General anesthesia was induced using remimazolam (6 mg/kg/h), rocuronium (0.9 mg/kg), and remifentanil (0.1 μg/kg/min) following sufficient pre-oxygenation. Sufficient mask ventilation could be obtained by the two-person technique. Endotracheal intubation using a 7.0 mm (ID) tube was performed using video laryngoscopy, and the tube was placed 16 cm from the right oral angle. While observing the tracheal lumen through the tube by bronchoscopy, the tube tip was placed just proximal to the narrowed region. The tube was fixed at a depth of 20 cm from the right oral angle. Her blood pressure and heart rate were controlled within ± 20% of the pre-induction values using intravenous nicardipine or landiolol during this procedure.

**Fig. 3:**
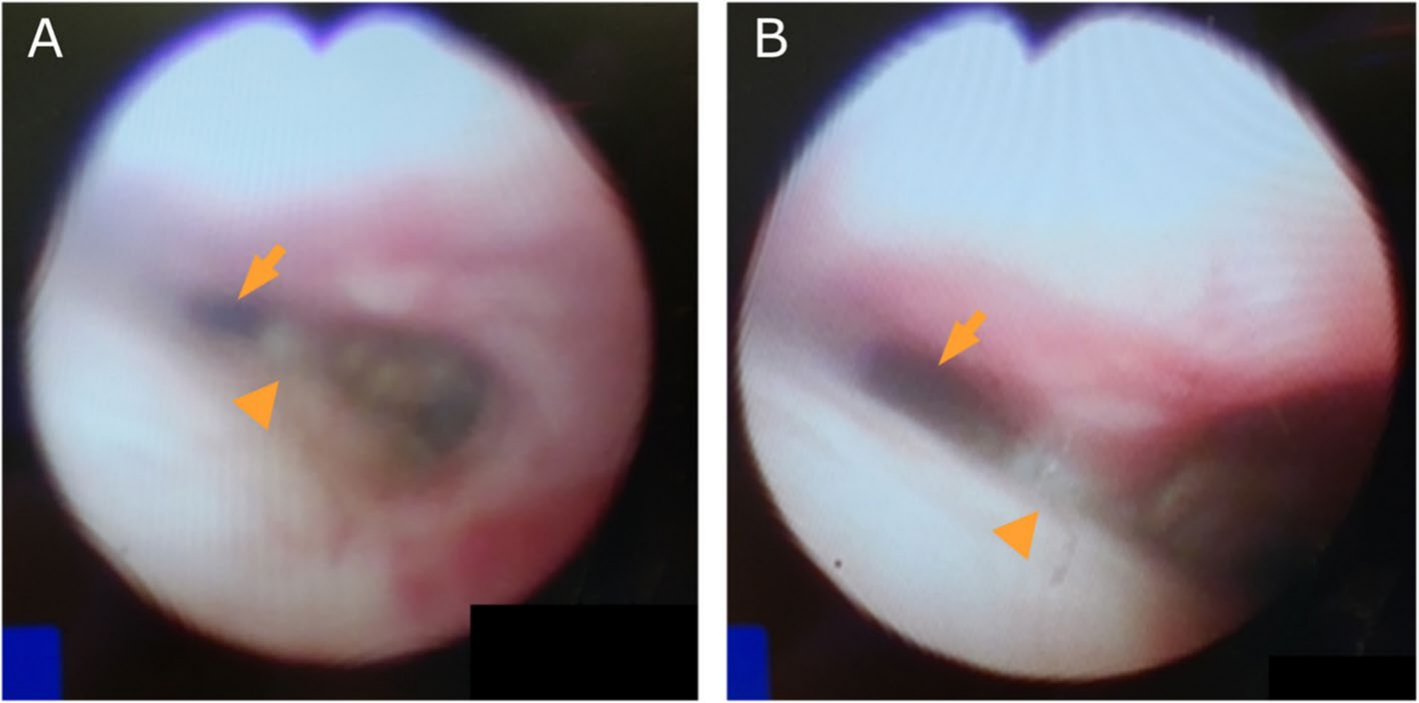
Image of bronchoscopy during the 2nd surgery. Tracheal lumen just after intubation for the second surgery (**A**). The signs of compression and stenosis of the trachea show improvement after TEVAR (**B**). Arrow: left trachea, arrowhead: carina

**Fig. 4:**
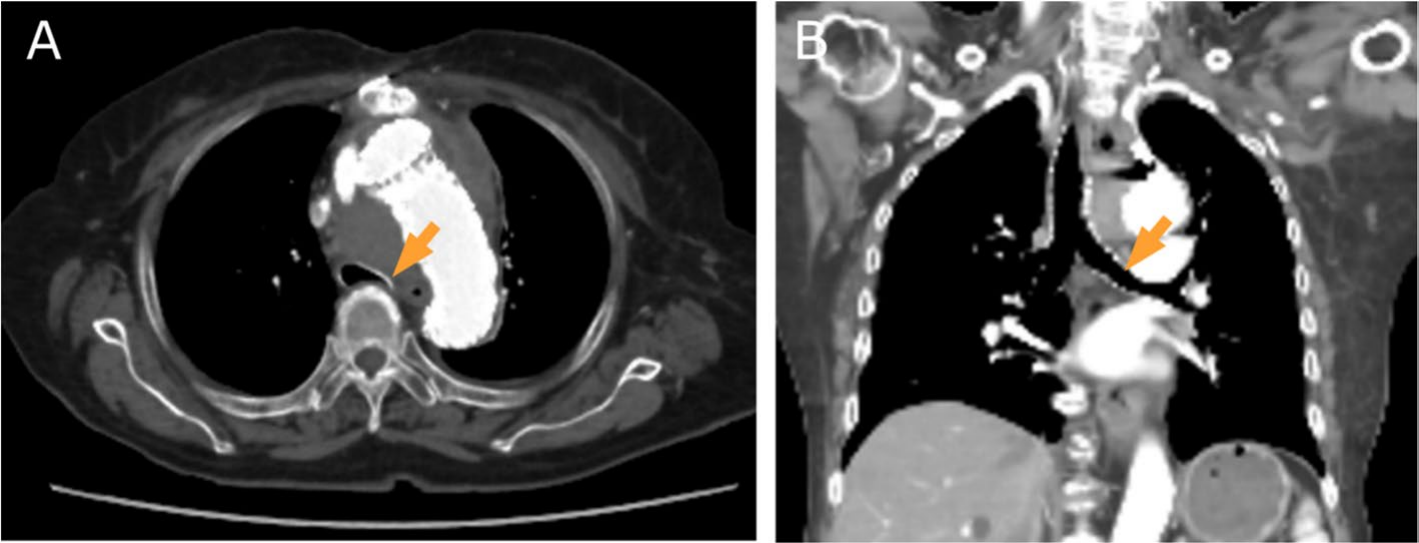
CT image of the chest on day Z + 7 (**A** transverse plane, **B** coronal plane). There is improvement of the tracheal compression (arrows)

Figure 3A shows the tracheal lumen after intubation. The signs of compression and stenosis of the trachea improved after TEVAR (Fig. 3B). The patient was awakened in the operating room and extubated. There were no significant problems with her respiratory status. CT of the chest on day Z + 7 showed improvement of the tracheal compression (Fig. 4).

## Discussion

We report a case of thoracic aortic aneurysm due to tracheal compression and stenosis that was discovered following ventilation difficulties. The treatment for the aneurysm was successfully conducted, and the risk of rupture was prevented.

To ensure the patient’s safety during the second surgery, it was necessary to avoid situations described as “cannot ventilate” and “can intubate - cannot ventilate” [1]. We also need to be cautious of the rupture of the aneurysm. To achieve the two goals, we planned general anesthesia with controlled ventilation. If we encounter a situation where we cannot ventilate before endotracheal intubation, we plan to position the patient in a right lateral position. To prevent a “can intubate - cannot ventilate” situation, we determined the depth of the tracheal tube under bronchoscopy. This procedure may also assist in preventing the rupture of an aortic aneurysm resulting from compression by the endotracheal tube. Since her oxygenation and ventilation were normal on room air, in cases where the tracheal tube was obstructed, like a check valve, it was considered to advance the tube carefully beyond the narrowed segment. If these do not work, we plan to reverse the effects of remimazolam and rocuronium with flumazenil and sugammadex to restore spontaneous breathing.

**Fig. 5:**
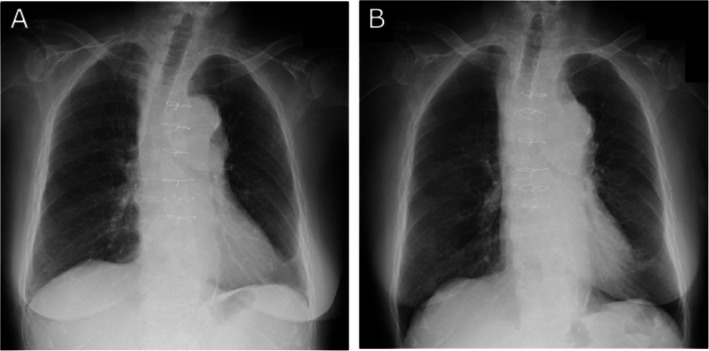
X-ray figure taken in upright position on the day Z−20 (**A**) and the day just before the first surgery (**B**)

Upper airway compression due to an aortic aneurysm usually presents with wheezing, coughing, dyspnea, or pneumonitis [2–6]. Our patient had been hospitalized for positional cough and was treated for heart failure 3 months before the first surgery. These symptoms may have been caused by dilation of the aneurysm; however, chest CT obtained 3 months before and X-ray figures taken on day Z−20 and on day Z immediately before the surgery in an upright position (Fig. 5) showed no clear findings indicative of re-enlargement of the aortic aneurysm and tracheal or bronchial stenosis. Prior to the induction of anesthesia, therefore, it was difficult to predict the presence of an aortic aneurysm that could cause ventilation difficulties. There are several case reports of aortic aneurysms that were found due to symptoms of dyspnea [6–10], but these studies identified airway narrowing due to an aortic aneurysm in imaging evaluations that were performed prior to the induction of anesthesia, and none reported the discovery of an aortic aneurysm during induction. In addition to the obstruction of the trachea itself, acute airway obstruction can also be caused by retropharyngeal hematoma due to a leaking aortic aneurysm [11].

This case lacks some preoperative assessments, such as re-evaluating chest CT scans closer to the time of surgery. If a patient with a history of aortic aneurysm experiences unexplained respiratory symptoms, an examination should be scheduled to evaluate the potential re-expansion of the aneurysm. Particular attention should be given to patients experiencing positional cough; they should consider using different diagnostic modalities in various body positions. Additionally, the establishment of extracorporeal circulation prior to anesthesia induction should be considered in cases of severe tracheal stenosis.

Compression from outside the trachea should be considered as a cause of sudden ventilation difficulties. Particular attention should be taken even in asymptomatic patients with a history of aortic disease.

## Data Availability

The data are not publicly available because of patient privacy concerns, but are available from the corresponding author on reasonable request.
